# Vaccine evaluation and genotype characterization in children infected with rotavirus in Qatar

**DOI:** 10.1038/s41390-023-02468-7

**Published:** 2023-01-19

**Authors:** Shilu Mathew, Hebah A. Al Khatib, Malak Al Ibrahim, Khalid Al Ansari, Maria K. Smatti, Gheyath K. Nasrallah, Emad Ibrahim, Asmaa A. Al Thani, Hassan Zaraket, Hadi M. Yassine

**Affiliations:** 1 Biomedical Research Center, Qatar University, 2713 Doha, Qatar; 2 Department of Experimental Pathology, Immunology, and Microbiology, American University of Beirut, Beirut, Lebanon; 3 Emergency Medicine Department, Sidra Medicine, Doha, Qatar; 4 College of Health Sciences-QU Health, Qatar University, 2713 Doha, Qatar; 5 Division of Clinical Microbiology, Department of Laboratory Medicine and Pathology, Microbiology Section, Hamad Medical Corporation, Doha, Qatar

## Abstract

**Background:**

We characterized and identified the genetic and antigenic variations of circulating rotavirus strains in comparison to used rotavirus vaccines.

**Methods:**

Rotavirus-positive samples (*n* = 231) were collected and analyzed. The VP7 and VP4 genes were sequenced and analyzed against the rotavirus vaccine strains. Antigenic variations were illustrated on the three-dimensional models of surface proteins.

**Results:**

In all, 59.7% of the hospitalized children were vaccinated, of which only 57.2% received two doses. There were no significant differences between the vaccinated and non-vaccinated groups in terms of clinical outcome. The G3 was the dominant genotype (40%) regardless of vaccination status. Several amino acid changes were identified in the VP7 and VP4 antigenic epitopes compared to the licensed vaccines. The highest variability was seen in the G3 (6 substitutions) and P[4] (11 substitutions) genotypes in comparison to RotaTeq®. In comparison to Rotarix®, G1 strains possessed three amino acid changes in 7-1a and 7-2 epitopes while P[8] strains possessed five amino acid changes in 8-1 and 8-3 epitopes.

**Conclusions:**

The current use of Rotarix® vaccine might not be effective in preventing the infection due to the higher numbers of G3-associated cases. The wide range of mutations in the antigenic epitopes compared to vaccine strains may compromise the vaccine’s effectiveness.

**Impact:**

- The reduced rotavirus vaccine effectiveness necessitate regular evaluation of the vaccine content to ensure optimal protection.
- We characterized and identified the genetic and antigenic variations of circulating rotavirus strains in comparison to the Rotarix vaccine strain that is used in Qatar.
- The study highlight the importance for regular monitoring of emerging rotavirus variants and their impact on vaccine effectiveness in young children.

## Introduction

Rotavirus (RV) is a leading cause of severe diarrheal infections among children under the age of 5 years. RV is estimated to cause 200K deaths and hundreds of thousands hospitalizations among children every year.^1,2^ Binary classification of RV is used to designate rotaviruses into G and P genotypes based on the genetic diversity of the capsid proteins, VP7 and VP4 segments, respectively.^3,4^ So far, 36 G and 51 P genotypes have been identified with G1, G2, G3, G4, G9, and G12 in combination with P[4], P[6], or P[8] being the most common genotypes associated with human infections.^5–8^

According to the World Health Organization (WHO) and Center of Disease Control (CDC), RV vaccination is the best way to protect against severe gastrointestinal disease.^9,10^ Four oral, live-attenuated RVA vaccines are currently available worldwide: Rotarix®, RotaTeq®, Rotavac®, and RotaSiil®. All four vaccines are approved by WHO and considered highly effective in preventing severe gastrointestinal disease among infected children (WHO). Rotarix® (RVA1) (GlaxoSmithKline, Brentford, United Kingdom) is a monovalent RV vaccine consisting of a single human G1P[8] strain.^11^ On the other hand, RotaTeq® (RVA5) (Merck & Co., Inc., United States), is a pentavalent human–bovine reassortant RV strain representing the most commonly circulating human RV genotypes (G1–G4 and P[8]). The implementation of RV vaccinations has subsequently lessened the burden of RV.^12–14^ However, the RV continues to evolve, necessitating continuous monitoring of the circulating strains worldwide.

In Qatar, RVA1 vaccine is administered to infants at 2 and 4 months of age. However, the vaccine use and effectiveness have not been assessed. In the present study, we estimated vaccine uptake among hospitalized children under 5 years of age with confirmed RV infections and analyzed the genetic diversity of the identified RV with respect to the vaccine strains.

## Materials and methods

### Sample collection

Samples were collected from children (<5 years old) visiting the Pediatric Emergency Center (PEC) of Hamad Medical Corporation (HMC) during 2016–2019 with gastrointestinal symptoms. Informed consent signed from the parents/legal guardians under IRB approval from HMC (Approval # 16173/19) and exemption from Qatar University (Approval # QU-IRB605-E/16) was obtained. Samples were collected from children within 48 h of their visit to the emergency center and followed up to 7 days. Demographical and clinical information were also collected. Collected clinical data included information about fever, duration and frequency of both diarrhea and vomiting, degree of dehydration, date of symptoms’ onset, admission and discharge dates, antibiotics and other treatments, RV vaccination, neurological symptoms, and underlying illnesses. Clinical data was used to evaluate the disease severity using Vesikari Clinical Severity Scoring System as described previously.^15,16^ Vesikari score was evaluated based on clinical manifestations developed during the 7-day follow-up period. In short, a score of <7 is considered mild, 7–10 is considered moderate, and >10 (up to 20) is considered severe.

### Viral nucleic acid extraction and RV genotyping

Stool samples were screened for RV using Film Array Gastrointestinal (GI) Panel kit (BIOFIRE®, Cambridge) at HMC. Positive samples were then transferred to the Biomedical Research Center (BRC) at Qatar University (QU) for further molecular characterization. Processing of fecal samples, RNA extraction and subsequent RV genotyping were performed as previously described.^16^ In short, 240 μL of fecal suspension supernatant was used for viral RNA extraction using QIAamp Viral RNA extraction kit (QIAGEN, Hilden, Germany) according to the manufacturer’s protocol. Purified viral RNA was then PCR amplified to determine G and P types. Full-length VP7 gene (810 bp) and partial VP4 fragment (630 bp) PCR products were checked using 1.5% agarose gel. PCR products were purified following the manual clean-up protocol (https://research.fredhutch.org/content/dam/stripe/hahn/methods/mol_biol/Agencourt%20AMPure%20XP.pdf). Sanger sequencing of all PCR products was performed at Macrogen (Seoul, Korea) using the PCR primers. The obtained sequences were utilized to determine the genotype using the web-based RotaC2.0 automated genotyping tool for rotaviruses.^17^ Multiple sequence alignments were done using CLUSTALW, and phylogeny trees were constructed with the Molecular Evolutionary Genetics Analysis Version 7.0 (MEGA 7) software using the neighbor-joining approach validated by replicating with 1000 bootstraps as previously reported.^18^

### Analysis of the VP4 and VP7 antigenic epitopes

To further investigate the variations within the antigenic epitopes of the circulating rotaviruses in Qatar, VP7 and VP4 gene sequences were compared with those included in RV vaccines: RVA1 (RVA/Vaccine/USA/Rotarix®;A41CB052A/1988/G1P1A[8]), RVA5 (RVA/Vaccine/USA/RotaTeq®-WI79-9/1992/G1P7[5], RVA/Vaccine/USA/RotaTeq®-SC2–9/1992/G2P7[5], G3 WI78-8/1992/G3P7[5]/RotaTeq® and RVA/Vaccine/USA/RotaTeq®-BrB-9/1996/G4P7[5]), and ROTAVAC.^10^ A mutation frequency (%) within each group (vaccinated vs non-vaccinated) was then calculated as the total number samples carrying any specific mutation/total number of samples within each group.

### Statistical analysis

Pearson’s chi-squared test was used to investigate significant differences between general categorical variables. All statistical data analyses were performed by GraphPad (Prism version 7) (IBM, Armonk, NY).

## Results

### Demographics and clinical manifestations of vaccinated and non-vaccinated RV- positive children

**Fig. 1:**
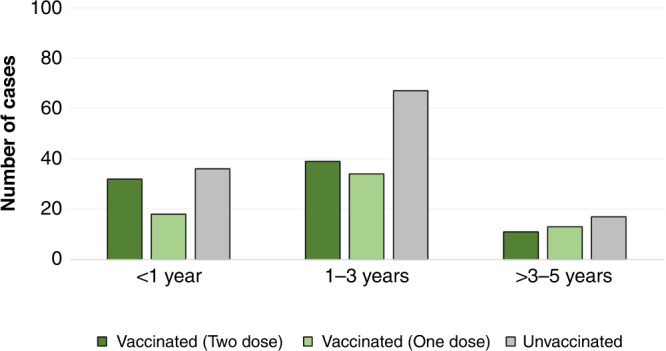
Distribution of RV-positive cases based on children’s age and RV vaccination status. *X*-axis denotes age in years and *Y*-axis denotes the number of infection cases.

We have previously reported a total number of 231 RV-positive children during the period from May 2016 to June 2019.^16^ Among the RV-positive children, 138 (60%) were vaccinated. Only 57.3% (*n* = 79) of age-eligible children received both doses of vaccine (Fig. 1). In terms of age, about 60% of children aged 0–12 months were vaccinated, followed by 56% of children aged 13–24 months, 73% of children aged 25–36 months, 62% of children aged 37–48 months, and 33% of children aged 49–60 months (Table 1).

**Table 1:** Demographics and vaccination status of RV-positive and RV-negative children included in this study.

Characteristics	Rotavirus-positive vaccinated cases	Vaccinated one dose	Vaccinated two doses	Rotavirus-positive non-vaccinated cases
	*n* = 138 (% of total RV cases)	*n* = 59 (% of vaccinated)	*n* = 79 (% of vaccinated)	*n* = 93 (% of total RV cases)
Age in months				
0–12 (*n* = 79)	45 (60%)	15 (33%)	30 (66%)	34 (40%)
13–24 (*n* = 66)	37 (56%)	17 (46%)	20 (54%)	29 (34%)
25–36 (*n* = 48)	35 (73%)	16 (45%)	19 (54%)	13 (27%)
37–48 (*n* = 29)	18 (62%)	9 (50%)	9 (50%)	11 (38%)
49–60 (*n* = 9)	3 (33%)	1	2	6 (67%)
Vaccination status				
Vaccinated one dose (*n* = 59)	59 (42.7%)	–	–	0
Vaccinated two doses (*n* = 79)	79 (57.3%)	–	–	0

**Fig. 2:**
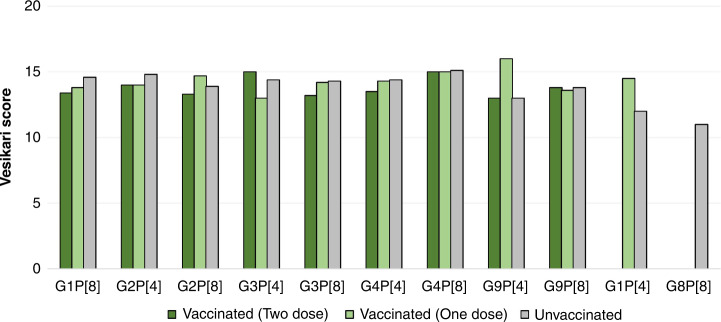
Association between RV genotypes and disease severity (Vesikari score) in vaccinated and unvaccinated children. *X*-axis denotes different circulating genotypes and *Y*-axis denotes Vesikari score.

Next, we assessed the association between vaccination status, RV genotype and infection severity. Infection severity was measured by Vesikari Clinical Severity Scoring System.^15^ Infection severity showed no significant differences among children regardless of their vaccination status or RV genotype (Fig. 2). The Vesikari score of infected children ranged from 13 to 16 in the one-dose vaccinated group, 13–15 in the two-dose vaccinated group and 11–15.1 in the non-vaccinated group (Fig. 2). The majority of RV-positive children—both vaccinated and non-vaccinated—experienced mild-to-moderate dehydration except for nine children, who suffered from severe dehydration and were treated with intravenous fluids (Table 2). Overall, non-vaccinated children experienced a relatively longer period of diarrhea (>5 days) with a higher frequency (4–7 times a day) compared to vaccinated children (Table 2). Only 10% of vaccinated children had diarrhea for >5 days compared to 19% of non-vaccinated children. Similarly, 27% of vaccinated children had higher diarrhea frequency compared to 35% of non-vaccinated children. Nonetheless, minimal difference was seen between the one-dose and two-dose vaccinated groups in terms of diarrhea duration (Table 2). In both groups, 75% of children had diarrhea for <3 days, while 10% had extended diarrhea duration that lasted for >5 days (Table 2). Extended vomiting periods (>5 days) at a higher frequency (five times a day) were observed among non-vaccinated children compared to vaccinated children. In terms of vomiting duration, 8% of non-vaccinated children had longer vomiting periods (>5 days) compared to 3% of vaccinated children. Similarly, 12% of non-vaccinated children experienced higher vomiting frequencies (>7 times a day) compared to vaccinated children (Table 2). Again here, no difference was detected between the one-dose and two-dose vaccinated groups in terms of vomiting duration and frequency (Table 2). Notably, 36.5% of non-vaccinated children had a long hospital stay (≥4 days) compared to 28.2% of vaccinated children. However, no significant difference between one-dose, two-doses, and non-vaccinated children in terms of the frequency and duration of vomiting and diarrhea (*P* value >0.05) was noted.

**Table 2:** Clinical symptoms and hospitalization details of vaccinated and non-vaccinated children infected with RV.

Clinical symptom/ treatment	Vaccinated (one dose)	Vaccinated (two doses)	Non-vaccinated
	*N* = 59 (%)	*N* = 79 (%)	*N* = 93 (%)
Dehydration			
No dehydration	0	0	0
Mild dehydration	35 (59.32%)	52 (65.82%)	50 (53.76%)
Moderate dehydration	22 (37.29%)	24 (30.4%)	39 (41.94%)
Severe dehydration	2 (3.39%)	3 (3.78%)	4 (4.3%)
Duration of diarrhea			
≤3 days	44 (74.58%)	60 (75.95%)	57 (61.3%)
3> and <5 days	9 (15.25%)	11 (13.92%)	18 (19.35%)
>5 days	6 (10.17%)	8 (10.13%)	18 (19.35%)
Frequency of diarrhea			
≤3	43 (72.88%)	57 (72.15%)	60 (64.52%)
>4 or equal to 7	16 (27.12%)	22 (27.85%)	33 (35.48%)
Duration of vomiting			
≤3 days	43 (72.88%)	51 (64.56%)	55 (59.14%)
>3 and <5 days	14 (23.73%)	25 (31.65%)	30 (32.26%)
>5 days	2 (3.39%)	3 (3.79%)	8 (8.6%)
Frequency of vomiting			
≤3	39 (66.1%)	23 (29.11%)	47 (50.54%)
>4 or equal to 7	16 (27.12%)	14 (17.72%)	34 (36.56%)
>7	4 (6.78%)	6 (7.59%)	12 (12.9%)
Hospital stays			
Short (<3 days)	39 (66.1%)	60 (75.95%)	59 (63.44%)
Long (>4 days)	20 (33.9%)	19 (25.05%)	34 (36.56%)

### The trends of RV genotypes among vaccinated and non-vaccinated children

**Fig. 3:**
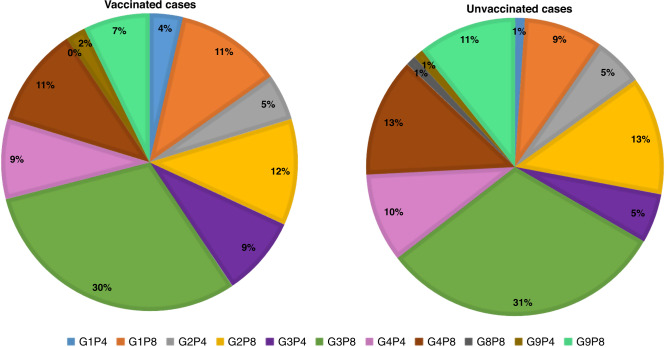
Prevalence of RV genotypes among vaccinated and unvaccinated RV-positive children.

Seasonal trends of RV infections in Qatar have been recently reported.^16^ Here, RV cases were reported throughout the year with a marginal increase during April and July of 2016–2019. As previously reported, six G (G1, G2, G3, G4, G8, and G9) and two P (P4 and P8) types were found among RV-positive children during 2016–2019.^16^ Overall, G3[P8] was the most prevalent genotype combination in all years regardless of vaccination status, accounting for 31% of cases in non-vaccinated children and 30% of cases in vaccinated children (Fig. 3). In fully vaccinated children, G2P[8] was the second prevalent with 12.3% (28/160), followed by G4P[8] with 11.6% (27/160), G1P[8] with 10.3% (24/160), and G4P[4] with 9% (21/160). The prevalence of RV genotypes was not different between one-dose and two-dose vaccinated children. In non-vaccinated children, G2P[8] was the second prevalent with 12.3% (28/160), followed by G4P[8] with 11.6% (27/160), G1P[8] with 10.3% (24/160), and G4P[4] with 9% (21/160**)** (Fig. 3). Low prevalent genotypes (2%-8%) including G9P[8], G3P[4], G2P[4], G1P[4], and G9P[4] were also reported in both vaccinated and non-vaccinated children. In addition, G1P[4] genotype was found in 4% of one-dose vaccinated and in 1% of non-vaccinated children but not in two-dose vaccinees. One case of G8, in combination with P[8], was detected among non-vaccinated children.

### Variations in VP7 antigenic epitopes

**Fig. 4:**
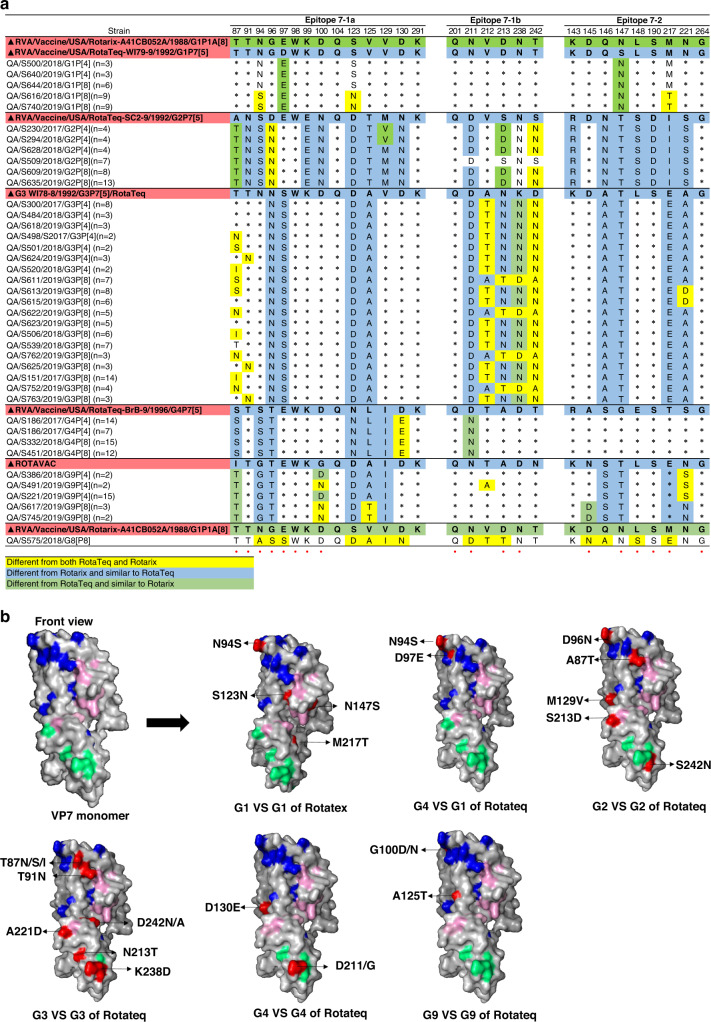
Antigenic changes in amino acid residues of VP7 epitopes. **a** Alignment of amino acid residues in VP7 antigenic sites of sequenced RV viruses against Rotarix and RotaTeq vaccines’ strains. Antigenic sites of VP7 are distributed in three main epitopes (7-1a, 7-1b, and 7-2). Residues that are different from strains in both Rotarix® and RotaTeq® are highlighted in yellow, residues that are different from the strains in RotaTeq® are highlighted in green, and residues that are different from strains in Rotarix® are highlighted in blue. Amino acid residues known to mediate escape from neutralization by mAbs are indicated by an asterisk (*). **b** Three-dimensional representation of amino acid substitutions detected in VP7 protein of RV strains. 3D structure of the VP7 monomer (gray color). Antigenic epitopes are colored in blue (7-1a), lime green (7-1b), and light pink (7–2). Surface-exposed residues that differ between circulating strains in Qatar and the strains contained in Rotarix or RotaTeq are shown in red color.

Next, we conducted a comparative analysis of antigenic epitopes of VP7 and VP4 in different RV genotypes compared to RVA1 and RVA5 vaccines. Alignment of amino acid sequences of the VP7 epitopes of the identified RV genotypes with the RVA1 and RVA5 vaccine strains revealed only four conserved amino acid residues among all the genotypes detected. Those residues are located at positions 98 and 104 in 7‐1a, 201 in 7‐1b, and 264 in 7‐2 antigenic sites. In all genotypes, variation sites in the VP7 region were mainly localized in 7-1a and 7-2 epitopes (Fig. 4). Among the G1 genotypes, 60% (*n* = 18) viruses harbored three amino acid substitutions (N94S, S123N, and M217T) in 7-1a antigenic site as compared to RVA1; and all had two substitutions (D97E, S147N) in 7-1a as compared to RVA5 vaccine (Fig. 4a). G2, G3, and G4 strains are not included in the RVA1 vaccine, therefore, VP7 epitopes of these genotypes were compared to RVA5 vaccine only. All G2 viruses possessed two amino acid substitutions: A87T and D96N in 7-1a antigenic sites compared to the G2 of the RVA5 vaccine. Three additional substitutions: M129V (7‐1a), S213D and S242N (7-1b) were found in 20% (*n* = 8), 82.5% (*n* = 33), and 82.5% (*n* = 33) of G2 genotype strains, respectively (Fig. 4a). Analysis of VP7 epitopes of G3 viruses revealed that 60.2% (n = 53/88) exhibited T87X substitution in the 7-1a epitope. Of these, 74% possessed the T87I/S substitution, while 26% had the T87N substitution (Fig. 4a). Other prevalent substitutions were found in 7-1b epitope of G3 viruses: A212T (78.4%) and D242A/N (100%). Less prevalent substitutions were also observed in G3 viruses including T91N (10.2%, *n* = 9/88) in 7-1a epitope; N213T (21.5%, *n* = 19/88) and K238D (22.7%, *n* = 20/88) in 7‐1b epitope, and A221D (16%, *n* = 14/88) in the 7‐2 epitope. All G4 genotyped viruses exhibited D130E and D211N substitutions in epitopes 7-1a and 7-1b, respectively. The G9 sequences were compared to both RAV5 and ROTAVAC vaccines. The latter is a G9 genotype vaccine that has been recently licensed in India. In comparison to RAV5, 29% and 21% of G9 strains had G100N/D and A125T substitutions in 7-1a epitope, respectively. In comparison to ROTAVAC, 79% of G9 viruses had the G100D substitution, while 100% had the I87T substitution within the 7-1a antigenic site (Fig. 4a, b). Additionally, N145D substitution was also detected in the 7-2 epitope of five G9 strain compared to ROTAVAC. The identified G8 virus showed amino acid differences in epitopes 7-1a (N94A, G96S, E97S, S123D, V125A, V129I, D130N), 7-1b (N211D, V212T, D213T), and 7-2 (D145N, Q146A, L148S, M217E) in comparison with RVA1 vaccine (Fig. 4a).

Overall, comparison of VP7 substitutions between vaccinated and unvaccinated children revealed limited number of significantly different substitutions. Six substitutions were found to be higher (*p* value <0.05) in unvaccinated children: T212A (G9), A212T (G3), N213T (G3), M217T (G1), N221S (G9), and D242A (G3) while one substitution in G9 strains, G100N, was found to be higher in vaccinated (Supplementary Fig. 1).

### Variations in VP4 antigenic epitopes

**Fig. 5:**
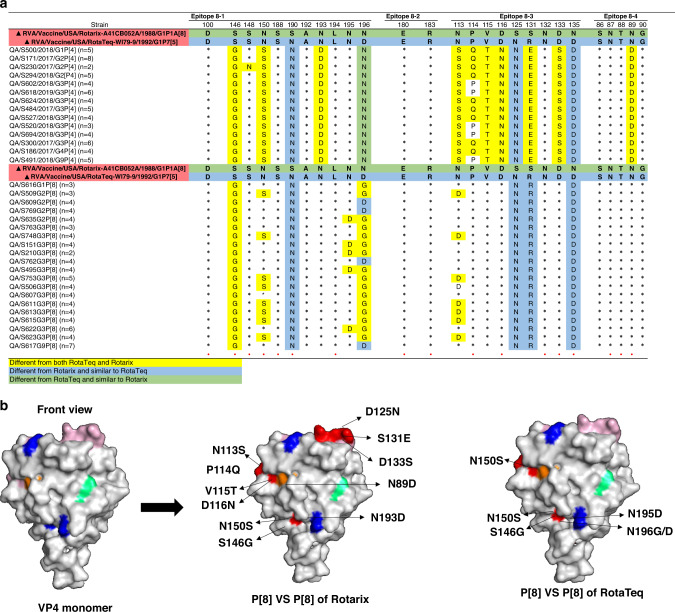
Antigenic changes in amino acid residues of VP4 epitopes. **a** Alignment of amino acid residues in VP4 antigenic sites of RV strains against Rotarix and RotaTeq vaccines’ strains. Antigenic sites of VP4 are distributed in four main epitopes (8-1, 8-2, 8-3, and 8-4). Residues that are different from strains in both Rotarix® and RotaTeq® are highlighted in yellow, residues that are different from the strains in RotaTeq® are highlighted in green, and residues that are different from strains in Rotarix® are highlighted in blue. Amino acid residues known to mediate escape from neutralization by mAbs are indicated by an asterisk (*). **b** Three-dimensional representation of amino acid substitutions detected in VP4 protein of RV strains. 3D structure of the VP4 monomer (gray color). Antigenic epitopes are colored in blue (8-1), lime green (8-2), light pink (8-3), and orange (8-4). Surface-exposed residues that differ between circulating strains in Qatar and the strains contained in Rotarix or RotaTeq are shown in red color.

The VP4 protein forms spikes on the virus surface and is implicated in virion attachment and entry in host cells. The VP8, which forms spike head of VP4, contains four surface-exposed neutralizing epitopes (8-1–8-4), and the VP5, the spike body of VP4, has five epitopes (5-1–5-5). Antibodies directed at VP8 epitopes were shown to neutralize RV infection by inhibiting viral attachment, and those against VP5 epitopes have been shown to block virion membrane penetration.^19^ Here, we evaluated the variation of antigenic region of VP8 by aligning 158 deduced amino acid sequences with the vaccine strains. The alignment revealed eight conserved amino acid residues (Fig. 5a). Overall, the VP8 antigenic epitopes of P[4] showed higher degree of variation compared to VP8 epitopes in P[8] strains. The differences were concentrated in VP4 8-1 and 8-3 (Fig. 5b). All VP8 antigenic epitopes of P[4] viruses exhibited 12 and 10 amino acid substitutions when compared to RVA1 and RVA5 vaccines, respectively. An additional prevalent amino acid substitution, P114Q, was found in P[8] strains of all G1, G2, G4 and 52% of G3 strains as compared to both vaccines. S148N substitution was exclusively found in 20% of P[8] strains in combination with G2 (Fig. 5a).

VP8 antigenic epitopes of P[8] viruses, on the other hand, showed higher degree of similarity with vaccine strains. All P[8] viruses exhibited six substitutions (S146G, S190N, N196D/G, S125N, S131R, and N135D) compared to RVA1 and one amino acid substitution (S146G) compared to RVA5 vaccines. Less prevalent substitutions were also found in positions 150 (epitope 8-1, 39%), 195 (epitope 8-1, 24%) and 113 (epitope 8-3, 34%) as compared to both vaccines (Fig. 5). Evaluating the prevalence of VP8 substitutions between vaccinated and unvaccinated children showed no significant differences between both groups. Only one substitution, P114Q in 8-2 epitope, was found to be higher in unvaccinated children infected with P[4] in combination with G3 and G9 genotypes (Supplementary Fig. 2).

## Discussion

Rotaviruses are common causes of acute gastroenteritis (AGE) in children worldwide, to which a vaccines are available.^20^ According to the CDC (WHO) guidelines, Rotarix® vaccine is given to children in two doses at ages 2 months and 4 months while RotaTeq® is given in three doses at ages 2 months, 4 months, and 6 months.^10^ Several post-licensure studies on RVA1 and RVA5 vaccines have demonstrated their safety and effectiveness in reducing RV-related hospitalizations among children.^21^ In April 2009, the WHO Strategic Advisory Group of Experts (SAGE) on immunization recommended the inclusion of RV vaccination of infants into all national immunization programs.^22^ Rotavirus vaccination strategy aims at reducing severe RV infections especially in countries where “diarrheal deaths account for ≥10% of mortality among children aged <5 years.^23,24^ Besides, it has been emphasized that the timely administration of RV vaccine doses should be accompanied by an informed immunization policy in the country. In Qatar, the RVA1 vaccine is available and is provided to children according to WHO vaccine recommendations. However, vaccine use, and efficacy have not been assessed in the country. In our recently published study, we investigated prevalence of RV genotypes and their seasonality trends in Qatar during 2016–2019. The same study has also investigated the association of different RV genotypes with age, gender, and nationalities of children.^16^ In this study, we compared RV infection severity and the prevalence of RV genotypes in vaccinated and non-vaccinated children. As expected, the majority of RV positive cases were reported among unvaccinated and one-dose vaccinated children. Despite the well-established pediatrics vaccination programs in Qatar, only 57.3% of age eligible children have received both doses of RVA1 vaccine. This could be attributed to the high number of expatriates in Qatar. We have previously shown that the majority of RV positive cases reported in Qatar during 2016–2019 belonged to children who came from low-income countries such Pakistani, Indian, Syrian and Yemini children where vaccination programs are poorly monitored.^16^ Notably, we showed that rates of disease severity were comparable between the vaccinated and unvaccinated children. The lack of significant differences in disease severity between both groups could be related to the monovalent nature of RVA1 vaccine. According to genotyping data, G3P[8] genotype was the most prevalent genotype among children in Qatar, regardless of their vaccination status. The RVA1 vaccine, on the other hand, consists of G1P[8] strain, which was detected in 9% and 11% of non-vaccinated and vaccinated children. This might suggest a lower effectiveness of vaccine and necessitate the importance of exploring other vaccine options. Despite the lack of significance in overall disease severity between vaccinated and non-vaccinated children, higher frequencies and longer periods of vomiting and diarrhea were reported among the non-vaccinated children. Similar findings were previously reported among RVA1 vaccinated children in 2016 in Brazil.^25^ The same study however, reported severe RV episodes among non-vaccinated and partially vaccinated children compared to fully vaccinated children.^25^ Other studies have also reported decreased disease severity and hospitalization rates among vaccinated children.^26^ The reported difference in vaccine effectiveness among these studies could be attributed to the economic status in any specific country. Decreased levels of vaccine effectiveness are usually reported in low-income countries where malnutrition and lack of sanitation is increasing the burden of disease.^27^

During 2016–2019, G3 genotype was the most prevalent in Qatar, accounting for 40% of RV-positive cases, followed by G2 (17.7%), G4 (16.8%), G9 (15%), G1 (9%), and G8 (0.9%). Based on VP4 genotyping, P[8] and P[4] were the most prevalent P genotypes in Qatar accounting for 70.6% and 29.4% of cases during 2016–2019.^16^ The G3P[8] was the most prevalent G/P combination (40%) in Qatar during 2016–2019, regardless of the vaccination status. Several studies have reported the association between G3 and P[8] genotypes in 50–65% of RV infections worldwide.^28,29^

G1P[8], which was detected in 9 and 11% of unvaccinated and vaccinated children, respectively, was the most prevalent RV genotype in many countries before the implementation of RV vaccine.^30–32^ Still, different G1P[8] sub-lineages are currently circulating, however, at lower frequency compared to G3 genotypes.^30^ A recent study studies have reported the emergence of specific G1P[8] clades among vaccinated RV-positive children, however, this was not associated with disease severity.^31,33^ Overall, we did not see major differences in the RV genotypes between vaccinated and unvaccinated groups. All genotypes were comparatively observed in both groups. The only exception was the G8P[8] genotype which was found in one unvaccinated children only.

Then, we compared the amino acid motifs constituting the neutralizing epitopes of VP7 and VP4 proteins in detected RV strains compared to vaccine strains. In total, 30 antigenic variations were spotted, 16 substitutions in VP7 and 14 substitutions in VP4. Several mutations were detected in the antigenic epitopes belonging to different genotypes. The G1 strains exhibited three prevalent substitutions in VP7: N94S, S123N, and M217T as compared to RVA1 vaccine. These substitutions were previously reported in vaccinated cohort from Australia,^34^ Belgium,^35^ Lebanon, Russia,^36^ India,^37^ and Pakistan.^38^ In this study, the three mutations were detected at comparable level in both vaccinated and unvaccinated children, suggesting a successful transmission of viruses that harbor these mutations. The S123N substitution was found at higher frequency among vaccinated children, which may suggest a potential immune selection. According to published data, however, S123N is not an immune escape mutation as demonstrated by monoclonal antibodies testing.^24^ On the other hand, mutations at 94 and 217 positions are known to be associated with immune escape.^39^ Pre-existing antibodies targeting 94 and 217 sites in VP7 of G1 strains can protect against RV infections.^29,40,41^ Therefore, the presence of N94S and M217T mutations is expected to compromise the neutralization properties of antibodies targeting VP7 antigenic sites.

Other genotypes have also exhibited several amino acid substitutions in VP7 epitopes. G3 strains carried the highest number of amino acid substitutions mainly located in 7-1b epitopes (*n* = 4 out of 7), with respect to RotaTeq® vaccine. This significant variation in G3 strains was previously seen in Italy, Lebanon, and Thailand.^8,30,42^ These major changes in G3 antigenic sites may explain their rapid local spread and might allow these strains to successfully spread globally in a relatively short period. All analyzed G2 strains have also carried three VP7 substitutions: A87T, D96N, and S213D. These mutations have been previously reported to be associated with vaccine introduction in different countries, possibly due to antigenic pressure of vaccine implementation.^29,43^ G2P[4] strains in our study exhibited two additional substitutions at positions 129 and 242. The latter was dominant among all G2 strains including G2P[4] and G2P[8], which might indicate a positive selection.^43^ Since RotaTeq vaccine is not used in Qatar, the detection of G2 and G3 mutations may suggest the introduction of these strains into the country, taking in consideration the multi-national nature of Qatar’s population. Emergence of these mutations could be also attributed to the presence of pre-existing immunity in previously infected children.^44,45^

The VP4 gene is more likely to be influenced by negative selection due to its key roles in attachment, penetration, and maturation. Therefore, it is reported to be less diverse compared to the VP7.^46,47^ However, in our study, we observed that antigenic variation in VP7 and VP4 was comparatively diverse to the vaccine strains. Analysis of the VP8 region of the VP4 gene revealed 14 substitutions compared to RVA1 and RVA5 vaccine strains. S146G, N150N, N193D, N113S, P114Q, V115T, D116N, and S131E are substitutions observed in the VP8 antigenic epitope. Amino acid substitution at positions 113, 114, 116, 146, and 150, in particular, are associated with escape from neutralizing antibodies.^48^ Overall, the emergence of antibody escape mutations in VP7 and VP4 antigenic sites may affect vaccine-acquired protection in vaccinated children.^28^ This underlines the importance of molecular epidemiological surveillance of circulating RV genotypes to monitor vaccine effectiveness and detect emergence of novel RV strains that may compromise vaccine-induced protection.

Despite these variations, though, the available RV vaccines are believed to be equally effective against the vast diversity of RV genotypes by generating heterotypic immune responses.^49^ Available vaccines have been shown to protect most cases from severe illness but not from RV infection.^50^ Multiple reviews from high and middle-income countries have reported a substantial reduction in disease burden within a few years of vaccine implementation through a decreased magnitude of RV-associated diarrhea and deaths.^42,51^

## Conclusion

Here, we investigated the prevalence of RV genotypes and infection severity in vaccinated and non-vaccinated children. Overall, our results demonstrated the dominance of the G3 genotype in all children regardless of vaccination status. The absence of G3 strain from the currently used monovalent Rotarix® vaccine may compromise vaccine-induced immunity. We have also reported high genetic variability between circulating G1 strains and Rotarix® G1 strain. This can further compromise vaccine effectiveness against G1 strains and hence underlines the importance of evaluating other vaccine options.

## Supplementary Information

Supplementary figures

## References

[1] Tate JE, Burton AH, Boschi-Pinto C, Parashar UD, World Health Organization–Coordinated Global Rotavirus Surveillance Network. (2016). Global, regional, and national estimates of rotavirus mortality in children <5 years of age, 2000-2013. Clin. Infect. Dis..

[2] Parashar UD, Hummelman EG, Bresee JS, Miller MA, Glass RI (2003). Global illness and deaths caused by rotavirus disease in children. Emerg. Infect. Dis..

[3] Knipe, D. M. & Howley, P. *Fields Virology*, Vol. 2 (LWW, 2012).

[4] Patton JT (2012). Rotavirus diversity and evolution in the post-vaccine world. Discov. Med..

[5] Leite JP, Carvalho-Costa FA, Linhares AC (2008). Group A rotavirus genotypes and the ongoing Brazilian experience: a review. Mem. Inst. Oswaldo Cruz.

[6] Iturriza-Gómara M (2011). Rotavirus genotypes co-circulating in Europe between 2006 and 2009 as determined by EuroRotaNet, a pan-European collaborative strain surveillance network. Epidemiol. Infect..

[7] Bányai K (2012). Systematic review of regional and temporal trends in global rotavirus strain diversity in the pre rotavirus vaccine era: insights for understanding the impact of rotavirus vaccination programs. Vaccine.

[8] Harastani HH (2020). Genetic diversity of human rotavirus a among hospitalized children under-5 years in Lebanon. Front. Immunol..

[9] World Health Organization. Immunization, vaccines and biologicals. https://www.who.int/teams/immunization-vaccines-and-biologicals/diseases/rotavirus (2021).

[10] CDC. Rotavirus vaccination. https://www.cdc.gov/vaccines/vpd/rotavirus/index.html (2018).

[11] Ward RL, Clark HF, Offit PA (2010). Influence of potential protective mechanisms on the development of live rotavirus vaccines. J. Infect. Dis..

[12] Ruiz-Palacios GM (2006). Safety and efficacy of an attenuated vaccine against severe rotavirus gastroenteritis. N. Engl. J. Med..

[13] Vesikari T (2007). Efficacy of human rotavirus vaccine against rotavirus gastroenteritis during the first 2 years of life in European infants: randomised, double-blind controlled study. Lancet.

[14] Jiang V, Jiang B, Tate J, Parashar UD, Patel MM (2010). Performance of rotavirus vaccines in developed and developing countries. Hum. Vaccines.

[15] Lewis, K. Vesikari clinical severity scoring system manual. PATH-a catalyst for global health. Version 1.3. https://media.path.org/documents/VAD_vesikari_scoring_manual.pdf (2011).

[16] Mathew, S., Al Ansari, K., Al Thani, A. A., Zaraket, H. & Yassine, H. M. Epidemiological, molecular, and clinical features of rotavirus infections among pediatrics in Qatar. *Eur. J. Clin. Microbiol. Infect. Dis*. 10.1007/s10096-020-04108-y (2021). 10.1007/s10096-020-04108-y 33411172

[17] Maes P, Matthijnssens J, Rahman M, Van Ranst M (2009). RotaC: a web-based tool for the complete genome classification of group A rotaviruses. BMC Microbiol..

[18] Tamura K (2011). MEGA5: molecular evolutionary genetics analysis using maximum likelihood, evolutionary distance, and maximum parsimony methods. Mol. Biol. Evol..

[19] Ludert JE, Ruiz MC, Hidalgo C, Liprandi F (2002). Antibodies to rotavirus outer capsid glycoprotein VP7 neutralize infectivity by inhibiting virion decapsidation. J. Virol..

[20] Elliott EJ (2007). Acute gastroenteritis in children. BMJ.

[21] Jonesteller CL, Burnett E, Yen C, Tate JE, Parashar UD (2017). Effectiveness of rotavirus vaccination: a systematic review of the first decade of global postlicensure data, 2006–2016. Clin. Infect. Dis..

[22] Organization WH (2009). Meeting of the immunization Strategic Advisory Group of Experts, April 2009—conclusions and recommendations. Wkly. Epidemiological Rec..

[23] Justino MCA (2011). Effectiveness of the monovalent G1P [8] human rotavirus vaccine against hospitalization for severe G2P [4] rotavirus gastroenteritis in Belém, Brazil. Pediatr. Infect. Dis. J..

[24] Salinas B (2005). Evaluation of safety, immunogenicity and efficacy of an attenuated rotavirus vaccine, RIX4414: a randomized, placebo-controlled trial in Latin American infants. Pediatr. Infect. Dis. J..

[25] Justino MCA (2016). Clinical severity and rotavirus vaccination among children hospitalized for acute gastroenteritis in Belém, Northern Brazil. J. Trop. Pediatr..

[26] Ono M (2020). Rotavirus genotype and Vesikari score of outpatients in Japan in the vaccine era. Pediatr. Int..

[27] Desselberger, U. Differences of rotavirus vaccine effectiveness by country: likely causes and contributing factors. *Pathogens*10.3390/pathogens6040065 (2017). 10.3390/pathogens6040065 PMC5750589 29231855

[28] George, S., Jagan, O. A., Bai, S. & Chandy, S. Genetic diversity of rotavirus strains in the era of vaccination: a pilot study from Central Kerala, India. *J. Clin. Diagn. Res.* **12**, DC01–DC06 (2018).

[29] Zeller M (2012). Genetic analyses reveal differences in the VP7 and VP4 antigenic epitopes between human rotaviruses circulating in Belgium and rotaviruses in Rotarix and RotaTeq. J. Clin. Microbiol..

[30] Bonura F (2022). Emergence in 2017-2019 of novel reassortant equine-like G3 rotavirus strains in Palermo, Sicily. Transbound. Emerg. Dis..

[31] da Silva MFM (2015). G1P[8] species A rotavirus over 27 years–pre-and post-vaccination eras–in Brazil: full genomic constellation analysis and no evidence for selection pressure by Rotarix(R) vaccine. Infect. Genet. Evol..

[32] Khoury H, Ogilvie I, El Khoury AC, Duan Y, Goetghebeur MM (2011). Burden of rotavirus gastroenteritis in the Middle Eastern and North African pediatric population. BMC Infect. Dis..

[33] Pradhan GN, Chitambar SD (2018). Full genomic analysis of G1P[8] rotavirus strains recovered from rotavirus vaccinated and non-vaccinated children hospitalized for acute gastroenteritis in Pune, western India. J. Med. Virol..

[34] Donato CM (2014). Characterization of a G1P[8] rotavirus causing an outbreak of gastroenteritis in the Northern Territory, Australia, in the vaccine era. Emerg. Microbes Infect..

[35] Zeller M (2012). Genetic analyses reveal differences in the VP7 and VP4 antigenic epitopes between human rotaviruses circulating in Belgium and rotaviruses in Rotarix and RotaTeq. J. Clin. Microbiol..

[36] Morozova OV, Sashina TA, Fomina SG, Novikova NA (2015). Comparative characteristics of the VP7 and VP4 antigenic epitopes of the rotaviruses circulating in Russia (Nizhny Novgorod) and the Rotarix and RotaTeq vaccines. Arch. Virol..

[37] Kulkarni R, Arora R, Arora R, Chitambar SD (2014). Sequence analysis of VP7 and VP4 genes of G1P[8] rotaviruses circulating among diarrhoeic children in Pune, India: a comparison with Rotarix and RotaTeq vaccine strains. Vaccine.

[38] Sadiq A, Bostan N (2020). Comparative analysis of G1P[8] rotaviruses identified prior to vaccine implementation in Pakistan with Rotarix™ and RotaTeq™ vaccine strains. Front. Immunol..

[39] Kirkwood CD, Bishop RF, Coulson BS (1998). Attachment and growth of human rotaviruses RV-3 and S12/85 in Caco-2 cells depend on VP4. J. Virol..

[40] Rippinger CM, Patton JT, McDonald SM (2010). Complete genome sequence analysis of candidate human rotavirus vaccine strains RV3 and 116E. Virology.

[41] Maranhão AG, Vianez-Júnior JL, Benati FJ, Bisch PM, Santos N (2012). Polymorphism of rotavirus genotype G1 in Brazil: in silico analysis of variant strains circulating in Rio de Janeiro from 1996 to 2004. Infect. Genet. Evol..

[42] Giaquinto C (2011). Summary of effectiveness and impact of rotavirus vaccination with the oral pentavalent rotavirus vaccine: a systematic review of the experience in industrialized countries. Hum. Vaccines.

[43] Thanh HD, Tran VT, Lim I, Kim W (2018). Emergence of human G2P[4] rotaviruses in the post-vaccination era in South Korea: footprints of multiple interspecies re-assortment events. Sci. Rep..

[44] Desselberger U, Huppertz HI (2011). Immune responses to rotavirus infection and vaccination and associated correlates of protection. J. Infect. Dis..

[45] Fischer TK (2002). Protective immunity after natural rotavirus infection: a community cohort study of newborn children in Guinea-Bissau, West Africa. J. Infect. Dis..

[46] Trask SD, McDonald SM, Patton JT (2012). Structural insights into the coupling of virion assembly and rotavirus replication. Nat. Rev. Microbiol..

[47] Enouf V, Chwetzoff S, Trugnan G, Cohen J (2003). Interactions of rotavirus VP4 spike protein with the endosomal protein Rab5 and the prenylated Rab acceptor PRA1. J. Virol..

[48] Estes M, Graham D, Mason B (1981). Proteolytic enhancement of rotavirus infectivity: molecular mechanisms. J. Virol..

[49] Alam MM (2013). Epidemiology and genetic diversity of rotavirus strains in children with acute gastroenteritis in Lahore, Pakistan. PLOS ONE.

[50] KidsHealth.org. Common questions about immunizations. https://kidshealth.org/en/parents/fact-myth-immunizations.html (2022).

[51] Patel MM, Clark AD, Sanderson CF, Tate J, Parashar UD (2012). Removing the age restrictions for rotavirus vaccination: a benefit-risk modeling analysis. PLoS Med..

