# Supplementary material for: Vaccine evaluation and genotype characterization in children infected with rotavirus in Qatar
Source: Pediatr Res. 2023 Jan 19;94(2):477–85. doi: 10.1038/s41390-023-02468-7 (PMC10382313; doi:10.1038/s41390-023-02468-7)
Supplement: Supplementary file 1 — Supplementary figures [file 41390_2023_2468_MOESM1_ESM.pdf]

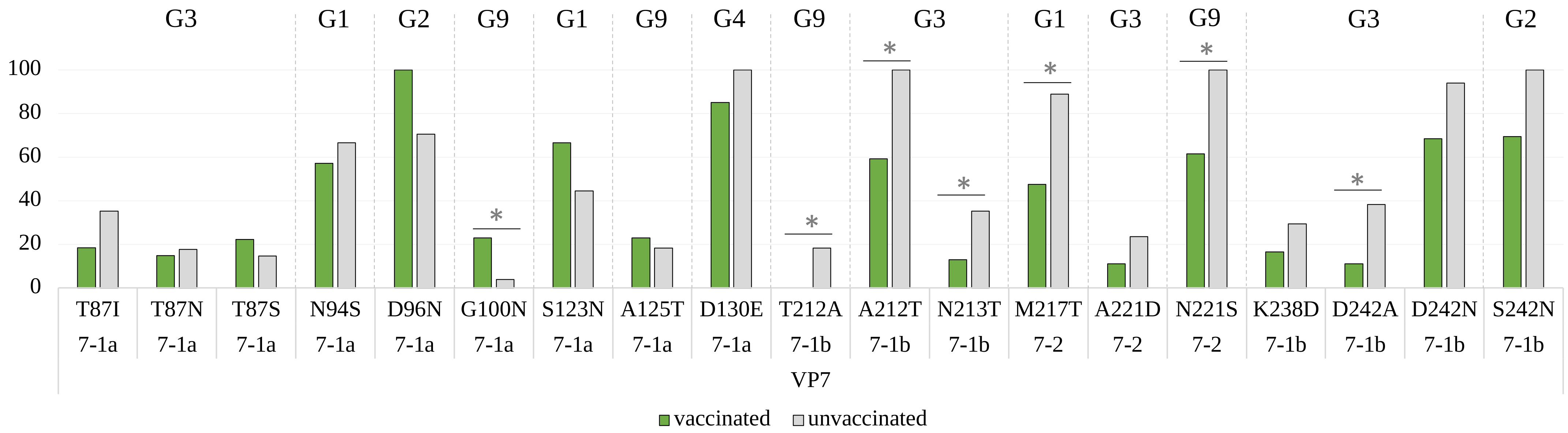

Supplementary figure 1: Prevalence of amino acid substitutions in antigenic epitopes of VP7 sequences. Chi-square test was used to calculate statistical significance. * indicates p-value of less than $p < 0.05$

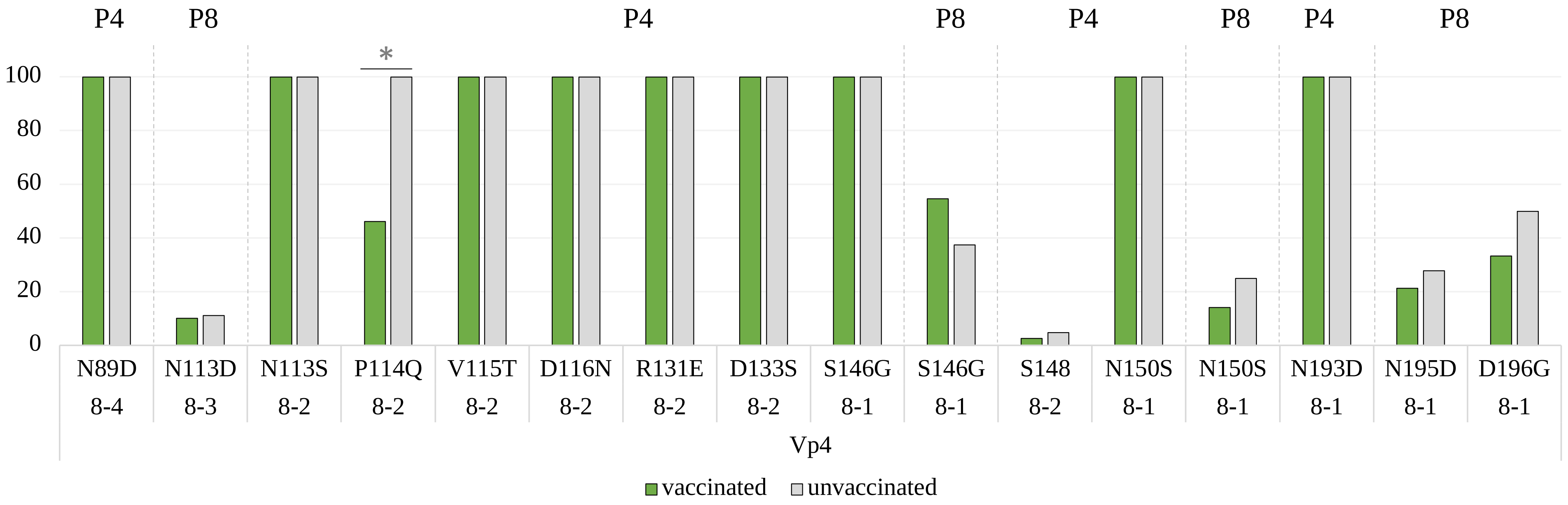

Supplementary figure 2: Prevalence of amino acid substitutions in antigenic epitopes of VP4 sequences. Chi-square test was used to calculate statistical significance. * indicates p-value of less than $p < 0.05$
